# Influence of Heat Events on the Composition of Airborne Bacterial Communities in Urban Ecosystems

**DOI:** 10.3390/ijerph15102295

**Published:** 2018-10-19

**Authors:** Zhiguo Fang, Weijun Guo, Junwen Zhang, Xiuqin Lou

**Affiliations:** 1 School of Environmental Science and Engineering, Zhejiang Gongshang University, Hangzhou 310012, China; mguoweijun@gmail.com (W.G.); zhangjunwen5561@163.com (J.Z.); 2 Hangzhou Center for Disease Control and Prevention, Hangzhou 310021, China; jolly3360@tom.com

**Keywords:** heat events, weather-dependent changes, airborne bacterial communities, bioaerosol

## Abstract

Airborne bacteria are significantly affected by meteorological and environmental conditions. However, there is little quantitative data available on the effects of these factors on airborne bacteria in urban ecosystems. In the present study, we analyzed weather-dependent changes in the composition of airborne bacterial communities using high throughput sequencing. Samples were collected before and after a period of constant hot weather at four selected sampling sites (YRBS, ZJGUSJC, TJCR, and BLQG) in Hangzhou. Our results show that the average amount of bacterial 16S rRNA gene copy numbers per m^3^ of air decreased significantly after constant high temperature. In addition, the number of operational taxonomic units and the Shannon–Wiener diversity indexes of the samples at all four selected sampling sites were significantly decreased after the heat event, showing notable impact on bacterial diversity. We also detected a significant increase in the abundances of spore-forming bacteria. Firmicutes increased from 3.7% to 9.9%, Bacillales increased from 2.6% to 7.6%, and Bacillaceae increased from 1.5% to 5.9%. In addition, we observed an increase in beta-Proteobacteria (18.2% to 50.3%), Rhodocyclaceae (6.9% to 29.9%), and Burkholderiaceae (8.1% to 15.2%). On the other hand, the abundance of alpha-Proteobacteria (39.6% to 9.8%), Caulobacteraceae (17.9% to 0.5%), Sphingomonadaceae (7.2% to 3.3%), and Xanthomonadaceae (3.0% to 0.5%) was significantly lower. Taken together, our data suggest that the composition of airborne bacterial communities varies greatly dependent on heat events, and that such communities include several species that are highly susceptible to high-temperature related stressors such as high air temperature, low relative humidity, and high intensity of solar radiation.

## 1. Introduction

Bioaerosols, which contribute as much as 25% to the atmospheric aerosols [1], are mixtures of viable and nonviable microbes (e.g., bacteria, fungi, yeasts, and algae) as well as other types of biomass, including a wide range of antigenic compounds, such as dander, plant and insect debris, microbial toxins, and viruses [2]. Bacteria found ubiquitously in the atmosphere, often originating from natural and anthropogenic sources, are the most important biogenic aerosol particles [1,3]. A conductive humid environment as well as many human activities, such as waterways and aeration [4,5,6], wastewater treatment [7,8], composting facilities [9], and solid waste and sewage transport and processing, may enhance the abundance of airborne bacteria in urban environments [10]. Exposure to bacterial aerosols is associated with a wide array of adverse health effects, and many bacterial pathogens such as *Streptococcus pneumoniae*, *Streptococcus pyogenes*, *Mycoplasma pneumoniae*, *Haemophilus influenzae*, *Klebsiella pneumoniae*, *Pseudomonas aeruginosa*, and *Mycobacterium tuberculosis* are often found in the air [11]. Apart from these pathogens, airborne microbial components (e.g., endotoxins, mycotoxins, and glucans) may also strongly influence human health and elicit allergic or inflammatory responses [11,12,13]. Furthermore, elevated bacterial concentration in the air is closely associated with the increasing probability of food pollution, deterioration of cosmetics and medicine, and corrosion of metallic materials for infrastructure [14,15].

These adverse effects on human health and direct or indirect influences on urban ecosystems have been driving forward research on airborne bacteria all over the world, and a general trend can be observed in different studies correlating the composition of bacterial communities and impact factors including meteorological conditions, environmental conditions, atmospheric composition, seasonality, anthropogenic influences, and variability in bacterial sources [3,11,15,16,17,18,19,20,21,22,23]. Jang et al. (2018) reported that heavy rainfall significantly affected the composition of airborne bacteria, and the abundances of airborne Propionibacteriaceae always increased after rainfall, whereas those of airborne Firmicutes consistently decreased [16]. In Milan (Italy), summer communities differed less from each other than the communities sampled during other seasons, since the stressors in summer, such as ozone, drought, and solar radiation all together induce a constant selective pressure leading consistently to the survival of adapted species [17]. At a set location, the wind, temperature, precipitation, and season affect the contribution of different source environments for airborne bacteria [3,18]. In Hong Kong, bioaerosol pollutants exhibited a strong seasonal trend related to general climatic variables, and were not significantly impacted by the level of urban development [19]. In urban environments, the composition of airborne bacteria in different areas is strongly affected by factors such as traffic conditions, human activities, and vegetation coverage [15]. Gao et al. (2016) reported the varying inter-relationship between the concentration and diameter of culturable bioaerosols and twelve factors in Beijing, and indicated that SO_2_ and NO_2_ show positive and negative correlations with culturable bioaerosol concentrations in the morning/evening and mid-day, respectively [20]. Recently, a number of studies have focused on bioaerosol composition during hazy and foggy days in China, due to heavy air pollution and frequent haze events [24,25,26,27]. However, the impact of heat event on airborne microbial communities remains poorly investigated.

China, like other places around the world, has experienced a significant warming trend since the mid-1980s. This trend is particularly pronounced in Southeast China, which has experienced a number of extreme heat events in recent years. Prolonged exposure to extremely high temperatures can lead to well documented health issues and has negative effects on many plant and animal species [28]. It may also lead to a lethal effects on bacterial physiology and structure [29,30]. The study of airborne bacterial communities during heat events is therefore especially important. Therefore, we chose Hangzhou as a model urban ecosystem to measure the changes in the composition of airborne bacterial communities before and after periods of extreme heat during the summer of 2013 in Zhejiang Province, Southeast China. Hangzhou, the capital and largest city of Zhejiang Province in China, has a subtropical monsoon climate, and is warm in the winter and hot in the summer with four distinctive seasons and abundant precipitation. The main objective of this study was to explore how extreme high temperature events influence the composition airborne bacterial communities, in particular, in the southeast urban area of China. We chose high-throughput sequencing of the 16S rRNA gene to determine changes in abundances of airborne bacterial species. The findings of this study might help to better understand and predict the structure and impact of airborne bacterial communities during heat events. 

## 2. Materials and Methods

### 2.1. Selection of Sampling Date

According to weather forecast from Zhejiang Meteorological Bureau in China (http://www.zjmb.gov.cn/zjqx/), 22 July 2013 was selected as the date sampling started (beginning the heat event) and 17 August 2013 was chosen as the finial sampling date (end the heat event), since the weather was thunder showers to cloudy on 21 July and cloudy to thunder showers on 18 August. In addition, relatively low air temperature (including maximum and minimum) from 1 to 21 July and 18 to 31 August 2013 was also found in Hangzhou. The weather condition of July and August 2013 was obtained from the network of weather report in China (http://www.tianqihoubao.com/lishi/hangzhou/month/201307.html and http://www.tianqihoubao.com/lishi/hangzhou/month/201308.html).

### 2.2. Description of Sampling Sites

Four typical sampling sites were selected for this study based on their urban function: (1) Tianmushan and Jiaogong Cross Road (TJCR)(30°16′17″ N, 120°8′12″ E), a heavily trafficked intersection located in Xihu district about 3 km from the city center; (2) Zhejiang Gongshang University Jiaogong Campus (ZJGSUJC) (30°17′7″ N, 120°8′20″ E), a cultural and educational area situated in Xihu district about 4 km from the city center; (3) Yan’an Road Business Street (YRBS) (30°15′45″ N, 120°9′50″ E), a commercial area and business district located at the center of Hangzhou city and in Xiacheng district; and (4) Breeze-ruffled Lotus at Quyuan Garden (BLQG) (30°14′55″ N, 120°7′57″ E), a scenic tourist area situated in Xihu district near West Lake, about 5 km from the city center. Detailed information about these selected sites can be found in Table 1.

### 2.3. Sample Collection

Air samples from selected sampling sites were collected using an air biocollector Coriolis μ air sampler (Bertin Technologies, Montigny-le-Bretonneux, France) presenting a cut-off size (d50) of 0.5 µm. The Coriolis μ air sampler is a continuous and high volume aerosol collection system, and the air is drawn into a conical vial in a whirling-type motion using suction. Airborne particles are pulled against the wall by centrifugal force, and are separated from the air and collected in a liquid medium. Samples of airborne bacteria were collected on a platform at a height of 1.5 m. Samples were collected in a sterile solution of 15 mL Tween 20 (Sigma, St. Louis, MO, USA) at 0.005% in milliQ water, and each sampling was carried out for 1.5 h at a calibrated flow rate of 0.25 m^3^ min^−1^ to obtain a total air sample volume of 22.5 m^3^. Due to evaporation, there are sample liquid losses at this flow rate. Therefore, the sterile solution was added up to 15 mL every 10 min during the sampling time. Sampling at the four selected sampling sites was conducted simultaneously with four Coriolis μ air samplers, and triplicate samples were taken from each selected sampling site at each date. The sampler head plate was thoroughly swabbed with 70% ethanol between sampling. Samples were stored on ice after sampling. 

### 2.4. Sample Concentration and DNA Extraction

Bacterial DNA extraction was performed by using QIAamp DNA Mini Kit (Qiagen, Hilden, Germany) according to the manufacturer’s instructions. In brief, samples were collected in 0.5% NaCl and transferred into a 15 mL sterile tube then centrifuged at 14,000× *g* for 10 min. The supernatant was concentrated using an Amicon Ultra Centrifugal Filter, Ultracel-100K (Billerica, MA, USA), and the final concentrated solute (60 µL) was mixed with the cell pellets collected in the first step. After resuspension, total DNA was extracted according to the instruction of isolation of genomic DNA from Gram-positive bacteria.

### 2.5. Quantitative PCR

The abundance of airborne bacteria was estimated by quantification of the copy number of the 16S rRNA gene. A 466 bp fragment of the bacterial 16S rDNA (331–797 according to *Escherichia coli* position, V3 and V4 hypervariable regions) was PCR-amplified with a universal primer set. The PCR was performed in a total volume of 30 μL reactions containing 15 µL of 2xSYBR^®^Green PCR Master Mix (Life Technologies, Carlsbad, CA, USA), 0.5 M Betaine (Sigma-Aldrich, Saint Louis, MO, USA), and V3–V4 specific 16S rRNA gene primers. The amplification was carried out under the following conditions 95 °C for 10 min, followed by 40 cycles of 95 °C for 30 s, 55 °C for 45 s, 72 °C for 45 s, followed by a melt curve analysis to ensure that primer-dimers were excluded from the analysis. Standard DNA dilution series were assayed in triplicate, and thermal cycling, data acquisition and analyses were carried out on an Applied Biosystems 7500 Real-Time PCR System with ABI 7500 Software v2.0.6 (Life Technologies, Carlsbad, CA, USA). To determine if the sample curves were significantly different from one another, the linear regression between Ct values versus log_10_ copies for each of four sets of standards was analyzed and compared to one another using the one-way analysis of variation (ANOVA) and the Bonferroni’s multiple comparisons test in GraphPad Prism5 software (GraphPad Software, San Diego, CA, USA).

### 2.6. PCR Amplification and Illumina Miseq PE300 Sequencing

The V3–V4 regions of the 16S rRNA gene were PCR-amplified for Illumina Miseq PE300 sequencing. Amplicon PCR was performed in 25 μL volume reactions with 12.5 μL of 2× KAPA HiFi HotStart ReadyMix, 2.5 μL of microbial DNA, and 5 μL of each primer (Klindworth et al., 2013) using primers 341 F (CCTACGGGNGGCWGCAG) and 805 R (GACTACHVGGGTATCTAATCC). Cycling conditions were as follows initial denaturation at 95 °C for 3 min, 25 cycles at 95 °C for 30 s, 55 °C for 30 s and 72 °C for 30 s, and a final extension at 72 °C for 5 min. At the 5′end of the 341 F primer, one of eight 8 bp barcodes was added to allow sample pooling and subsequent sequence sorting. The amplified products of 550 bp were purified with AMPure XP beads to exclude free primers and primer dimer species. Index PCR was performed in 50 μL volume reactions with 25 μL of 2× KAPA HiFi HotStart ReadyMix, 5 μL of DNA, 5 μM of primer 1, and index primer (8 bp barcodes). Cycling conditions were as follows initial denaturation at 95 °C for 3 min; 8 cycles at 95 °C for 30 s, 62 °C for 30 s, and 72 °C for 30 s and a final extension at 72 °C for 5 min. DNA quantity and purity was evaluated spectrophotometrically using NanoDrop 2000 (Thermo Scientific, Waltham, MA, USA), and the DNA samples were sequenced at XY Biotechnology Co. Ltd. (Shanghai, China) using paired-end sequencing on a Illumina MiSeq PE300 platform.

### 2.7. Sequence Analysis

QIIME v.1.8.0 software (Caporaso et al., Denver, CO, USA) was used to trim all pyrosequencing reads. After trimming, the reads that were shorter than 200 bp, contained mismatches and ambiguous bases (N) in primer, or exhibited a homopolymer longer than 10 bp were removed. All samples were normalized to the same sequencing depth using Mothur v.1.30.1 software (Schloss et al., Washtenaw, MI, USA). The operational taxonomic units (OTUs) were clustered with a 97% similarity cutoff using Usearch v. 5.2.236 (Edgar, Tiburon, CA, USA), and the chimeric sequences were identified and removed using UCHIME v. 4.2.40 (Edgar et al., Tiburon, CA, USA). The Alpha diversity of samples, mainly of the Shannon index, were determined using Mothur v.1.30.1. Taxonomic classification at the phylum and genus level was performed using the Ribosome Database Project (RDP) algorithm to classify the representative sequences of each operational taxonomic unit (OTU). The relative abundance of each phylum, class, order, family, and genus was calculated by comparing the number of sequences classified as the phylum, class, order, family, and genus to the number of total bacterial 16S rDNA gene sequences detected per sample.

### 2.8. Statistical Analysis

All experimental data were analyzed with Excel 2010 and SPSS Version 19.0 (Standard Version, SPSS Inc., Chicago, IL, USA), and one-way analysis of variance (ANOVA). The significance level was set to 0.05.

## 3. Results

### 3.1. Weather Conditions during Sampling

**Figure 1:**
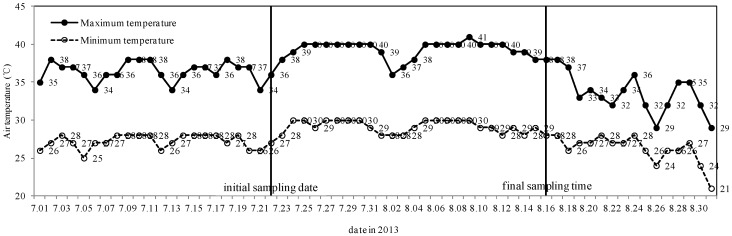
Air temperature chart of Hangzhou during July and August in 2013.

Hangzhou had 18 sunny days, three sunny to cloudy days, two cloudy to sunny days, and four cloudy days with no rainy days from 22 July to 17 August 2013 (Figure 1). The average maximum and minimum air temperatures daily between 22 July 2013 and 17 August 2013 were 39.1 and 29.0 °C, respectively. Over this period, maximum temperatures during the day varied between 41 (one day), 40 (14 days), 39 (four days), 38 (five days), 37 (one day), and 36 °C (two days). However, the average maximum and minimum air temperatures daily were 36.4 and 27.2 °C, respectively, from 1 July to 21 July, and 33.4 and 26.4 °C from 18 August to 31 August, respectively, significantly lower than during the high temperature period.

### 3.2. 16S rRNA Gene Analysis of Airborne Bacteria

**Figure 2:**
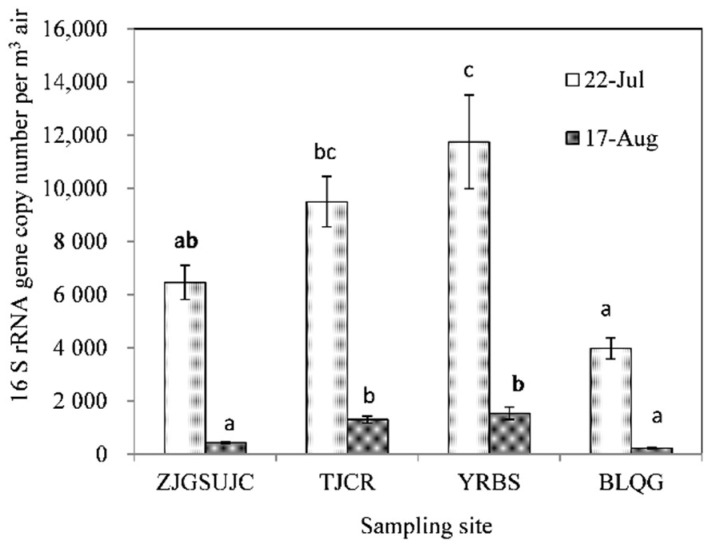
Airborne bacterial 16S rRNA gene copies as determined by quantitative real-time PCR beginning and end the heat event at four selected sampling sites in Hangzhou.

The abundance of airborne bacteria was estimated by the quantification of the number of 16S rRNA gene copies. Airborne bacteria from four selected sampling sites before and after a period of extreme heat were quantified by total DNA extracts. The standard curve was linear from 2 × 10^0^ to 2 × 10^5^ gene copies per reaction (r^2^ = 0.998). Averages of 7.9 × 10^3^ and 8.8 × 10^2^ bacterial 16S rRNA gene copies per m^3^ of air were determined on 22 July 2013 and 17 August 2013, respectively, and significantly higher 16S rRNA gene copy numbers were detected after the heat event (*p* < 0.01) (Figure 2). The bacterial 16S rRNA gene copy numbers were approximately 6.5 × 10^3^, 9.5 × 10^3^, 1.2 × 10^4^, and 4.0 × 10^3^ per m^3^ of air at the sampling sites of ZJGSUJC, TJCR, YRBS, and BLQG, respectively, at the start of the heat event. At the end of the heat event, bacterial 16S rRNA gene copy numbers were approximately 4.4 × 10^2^, 1.3 × 10^3^, 1.5 × 10^3^, and 2.3 × 10^2^ per m^3^ of air at the sampling sites of ZJGSUJC, TJCR, YRBS, and BLQG, respectively. Taken together, 16S rRNA gene copy numbers at the selected four sampling sites were significantly higher before the heat event (*p* < 0.01).

### 3.3. OTUs (Operational Taxonomic Units) and Shannon–Wiener Diversity Index

**Figure 3:**
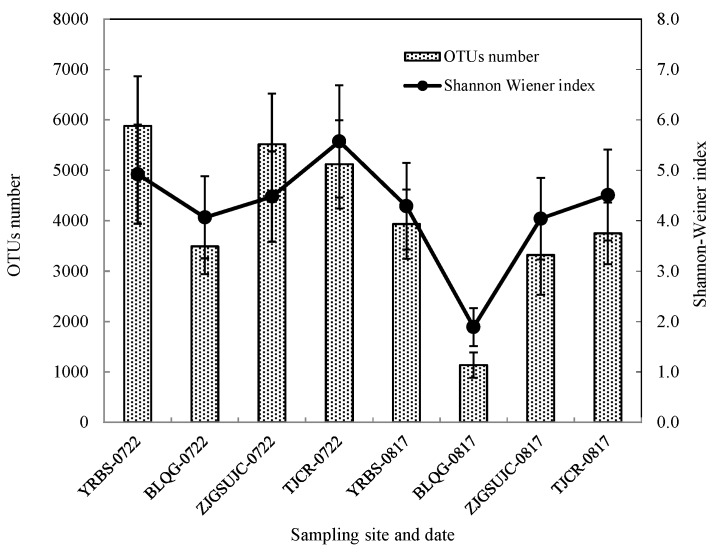
OTUs and Shannon–Wiener index of airborne bacteria before and after heat events.

Number of OTUs and Shannon–Wiener indexes of the analyzed airborne bacterial communities before and after the heat event are shown in Figure 3. For all four selected sampling sites, OTUs numbers and Shannon–Wiener indexes were significantly higher before the period of extreme heat (*p* < 0.05), indicating that bacterial diversity was reduced after extreme weather.

### 3.4. Abundance of Bacterial Phyla in Airborne Bacterial Communities

**Figure 4:**
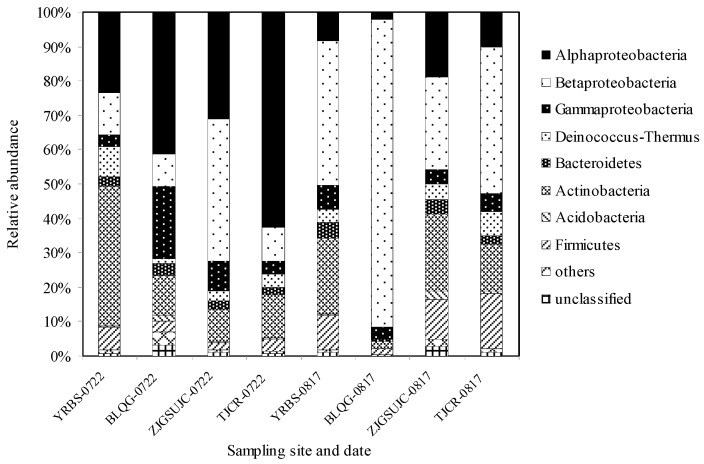
Types and abundance of airborne bacteria at Phylum taxonomic levels before and after heat events.

Overall, we observed that the air samples collected on prior to the heat event (22 July 2013) were dominated by Proteobacteria (67.1%), Actinobacteria (18.4%), Deinococcus–Thermus (4.1%), Firmicutes (3.7%), and Bacteroidetes (2.8%). In contrast, the predominant bacterial phyla of the samples collected after the heat event (17 August 2013) were Proteobacteria (65.1%), Actinobacteria (15.1%), Firmicutes (9.9%), Deinococcus–Thermus (3.9%), and Bacteroidetes (2.9%). Compared to the samples before the period of extremely high temperature, the proportion of Proteobacteria, Actinobacteria, and Deinococcus–Thermus decreased slightly, while the proportion of spore-forming bacteria such as Firmicutes increased significantly. We also found that the proportion of alpha-Proteobacteria and gamma-Proteobacteria was significantly higher on before the heat event (*p* < 0.01), while the proportion of beta-Proteobacteria was significantly lower (*p* < 0.01). The abundances of alpha-, beta-, and gamma-Proteobacteria were 39.6%, 18.2%, and 9.3% before the heat event, and changed to 9.8%, 50.3%, and 5.0% after the heat event, respectively (Figure 4).

### 3.5. Dominant Bacterial Orders before and after the Heat Event

**Figure 5:**
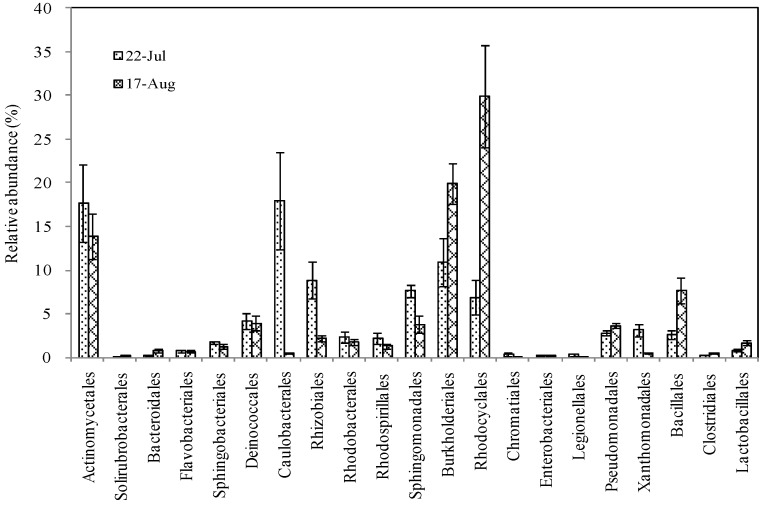
Types and abundance of dominant airborne bacteria at Order taxonomic levels before and after heat events.

The prevalent bacterial groups of air samples collected before the heat event were Caulobacterales (17.9%), Actinomycetales (17.7%), Burkholderiales (10.9%), Rhizobiales (8.9%), Sphingomonadales (7.7%), and Rhodocyclales (6.9%). After the heat event, Rhodocyclales (29.9%), Burkholderiales (19.9%), Actinomycetales (13.9%), and Bacillales (7.6%) were most abundant (Figure 5). Compared to the samples before the period of extreme heat, Burkholderiales, Rhodocyclales, and Bacillales were significantly more abundant after the heat event (*p* < 0.05), and the proportion of Rhodocyclales increased dramatically from 6.9% to 29.9%, Burkholderiales from 10.9% to 19.9%, and Bacillales from 2.6% to 7.6%. In addition, Actinomycetales, Caulobacterales, Rhizobiales, Sphingomonadales, and Xanthomonadales were significantly less abundant after the heat event (*p* < 0.05), and the abundance of Caulobacterales decreased dramatically from 17.9% to 0.5%, Actinomycetales from 17.7% to 13.9%, Rhizobiales from 8.9% to 2.2%, Sphingomonadales from 7.7% to 3.9%, and Xanthomonadales from 3.1% to 0.5%.

### 3.6. Bacterial Families Differentiate after a Period of Extreme Heat

In general, the predominant bacterial families before the heat event were Caulobacteraceae (17.9%), Burkholderiaceae (8.1%), Sphingomonadaceae (7.2%), Rhodocyclaceae (6.9%), Deinococcaceae (4.0%), and Xanthomonadaceae (3.0%). After the heat event, Rhodocyclaceae (29.9%), Burkholderiaceae (15.2%), Bacillaceae (5.9%), Deinococcaceae (3.9%), Sphingomonadaceae (3.3%), and Comamonadaceae (2.7%) were prevalent (Table 2). Compared to the samples before the period of extremely high temperature, we detected a significantly higher proportion of Bacillaceae, Burkholderiaceae, Comamonadaceae, Moraxellaceae, and Rhodocyclaceae at 17 August 2013 (*p* < 0.05), and the proportion of Rhodocyclaceae increased from 6.9% to 29.9%, Burkholderiaceae from 8.1% to 15.2%, Bacillaceae from 1.5% to 5.9%, Comamonadaceae from 1.5% to 2.7%, and Moraxellaceae from 1.5% to 2.4%. In contrast, we detected a significantly lower proportion of Bradyrhizobiaceae, Caulobacteraceae, Micrococcaceae, Rhodospirillaceae, Sphingomonadaceae, and Xanthomonadaceae after the heat event (*p* < 0.05), and the proportion of Caulobacteraceae decreased dramatically from 17.9% to 0.5%, Sphingomonadaceae from 7.2% to 3.3%, Xanthomonadaceae from 3.0% to 0.5%, Micrococcaceae from 2.8% to 1.3%, Rhodospirillaceae from 1.4% to 0.3%, and Bradyrhizobiaceae from 1.3% to 0.1%. Finally, Microbacteriaceae (from 1.9% to 1.2%) and Rhodobacteraceae (from 2.4 to 1.8%) were slightly less abundant.

## 4. Discussion

Our results showed that the heat event in Hangzhou significantly affected the composition of the airborne bacterial communities. During the heat event with no rainy day from 22 July 2013 to 17 August 2013, the weather of Hangzhou was characterized by high air temperature, low relatively humidity, and high intensity of solar radiation at the atmosphere. Airborne bacteria have different distribution regarding their optimal living temperatures, and air temperature can promote or restrain bacterial release and growth. With an analogous function such as temperature, relative humidity has a significant impact on airborne bacterial diversity and composition, and effects combining with temperature are most likely to be produced. Additionally, solar radiation may provide an unfavorable condition for the growth and survival of airborne bacteria and can kill these bacteria because of the microbicidal efficacy of UV [31]. Many studies also confirmed that bacterial communities in the air were strongly affected by those meteorological factors. Zhen et al. (2017) reported that meteorological factors such as air temperature, relative humidity, etc. had more impact on airborne bacterial communities than air pollutants [32]. In Upper Silesia, Poland, the most important meteorological factors related to the viability of airborne bacteria were temperature, UV radiation, and relative humidity [33]. It was also reported that the significant influences on airborne bacteria and pigment organisms at the rural sites were temperature, relative humidity, solar radiation, etc. [34]. Hoeksma et al. (2015) studied the effects of temperature on *Escherichia coli*, *Enterococcus mundtii*, and *Mycoplasma synoviae* and observed that temperature assuredly had significant influence on bacteria [35]. Mouli er al. (2005) reported that high relative humidity may also reduce the viability of bacteria [36]. Tong et al. (1997 and 1998) reported that high solar radiation had a lethal effect on airborne bacteria, and the degree of lethality correlated with the intensity, time, and wavelength of the light [22,37]. The effect of solar radiation on bacterial survival was also investigated by Tong et al. (1997), and bacteria collected on sunny days were preselected for their survivability [37]. These results show that meteorological factors have significantly positive or negative effects on the bacterial composition in the air. Therefore, bacterial communities in Hangzhou varied greatly after the heat event with the above mentioned weather characteristics.

In the present study, the relative abundances of Firmicutes, Bacillales, and Bacillaceae all increased significantly after the heat event. As we known, Firmicutes, Bacillales, and Bacillaceae have a Gram-positive cell wall structure and many of them can produce spores, which are resistant to desiccation and can survive in extreme environments. Jung et al. (2009) investigated the thermal effects on bacterial bioaerosols of *Escherichia coli* and *Bacillus subtilis*, and their results showed that Gram-negative bacteria are more susceptible to damage than Gram-positive bacteria in conditions of thermal stress caused by high surrounding temperature [30]. Ray et al. (1984) reported that Gram-positive cells have a fairly rigid and protective shell membrane wall, which may provide better protection against structural injury than metabolic injury during exposure for short periods at high temperatures [38]. For instance, the cell envelope of Gram-positive bacteria is similar to a thick insulator that protects the cell against changes in the environment, while the Gram-negative bacteria cell envelope acts like a thin insulator, which offers considerably less protection against adverse environmental conditions [39]. Once the environment is not favorable for bacterial growth, the protective mechanisms of some bacteria will be induced, which results in the survival of adapted bacterial species in extreme environments. Such protective mechanisms include DNA repair mechanisms, pigmentation, mechanisms promoting aggregation, and metabolic adaptations to nutrient shortages, which all contribute to survival [40]. Another protective mechanism of bacteria is to enter a nondividing state (dormancy), where bacteria morphologically transform to spores, undergo other cell wall modifications, and slow-down or stop their metabolic activity [41]. These transformations can improve the resistance to physical stressors, which increases the chance of survival in the atmosphere [42]. Similar to our finding that the abundance of Bacillales increases after the heat event, the viable fraction of airborne bacteria often seems to consist mainly of spore-forming bacteria after Asian dust events [43]. Since the atmosphere during an extreme heat event is a very harsh environment for bacteria, it is not surprising that the proportion of spore-forming bacteria that can tolerate extreme environments increased sharply, and the relative abundance of Caulobacteraceae decreased significantly after the heat event. However, the relative abundance of Rhodocyclaceae and Burkholderiaceae increased significantly, the impact of the heat event on the viability of those bacteria in the air is not fully clear and should be further studied. 

The number of OTUs as well as the Shannon–Wiener indexes of the samples were decreased, which suggested that bacterial diversity at the atmosphere was reduced after the heat event in Hangzhou. Simultaneously, 16S rRNA gene copy numbers at the selected four sampling sites were significantly lower after the heat event. This meant that bacterial concentrations in the air were also dramatically affected by air temperature, relatively humidity, and solar radiation. Higher temperature related to strong ultraviolet radiation are not suitable for the propagation and growth of bacteria and lead to the denaturation and inactivation of proteins. Tang (2009) also showed that temperatures above approximately 24 °C appeared to universally decrease airborne bacterial survival due to protein inactivation caused by high temperature [44]. Wu et al. (2012) reported that air temperature and relative humidity were correlated with total bacteria and Gram-negative cocci, respectively [45]. It was also demonstrated by Di Giorgio et al. (1996) that air temperature and relative humidity had a positive effect on the viability of airborne bacteria, and bacterial concentration increased with the rise of air temperature and relative humidity in a certain range [46]. At urban, rural and coastal sites, and forest areas, the total amount of airborne bacteria was significantly influenced by relative humidity, and bacterial levels in the air increased with relative humidity [34,47]. Liu et al. (2018) reported that relative humidity etc. was the main factor affecting the diversity of total bacteria and the proportion of pathogenic bacteria [48]. In a desert location, it was observed that bacterial concentrations were reduced at noon when destruction by solar radiation was greatest. The bacterial density in samples sampled with coverage was significantly higher than in those sampled with no coverage, and solar radiation could reduce the amount of bioaerosol generated from soil [49]. Fang et al. (2007) also reported that airborne bacteria were damaged by UV radiation with the gradually increasing radiation [15]. Generally, some bacteria have high abundance at high temperature, some at low, but most of them occurred at moderate values of the air temperature, and appropriate temperature can facilitate the release and growth of bacteria so that it can lead to the increase of bacterial concentration. In our study, a continuous and extreme heat event in Hangzhou had negative effects on the survival of airborne bacteria, and led to a great decrease of bacterial density in the atmosphere.

Finally, many studies also showed that wind speed and direction played an indispensable role in affecting ambient bacterial characteristics [31,50]. In this study, the potential importance of wind direction as an unconstrained factor could also influence the sources and abundance patterns of airborne bacteria. However, our data in the present study are very interesting, and patterns were consistent across four sampling sites even in the absence of full meteorological conditions and, even provided that a lack of information on wind direction is acknowledged as a limitation, the study is still a useful contribution. 

## 5. Conclusions

We analyzed the density and composition of airborne bacterial communities at four selected sampling sites (YRBS, ZJGUSJC, TJCR, and BLQG) in Hangzhou before and after a heat event. We drew multiple conclusions from our data: (i) The concentration of bacteria in the atmosphere was greatly affected by constant extremely high temperature, and we observed a significant decrease of, on average, 7.9 × 10^3^ to 8.8 × 10^2^ bacterial 16S rRNA gene copies per m^3^ beginning and end the heat event. (ii) The number of OTUs and the Shannon–Wiener indexes of the samples of airborne bacteria decreased, indicating that bacterial diversity was reduced after extreme weather. (iii) The composition of the airborne bacterial communities varied greatly, and the abundance of spore-forming bacteria increased significantly after the heat event. 

## Figures and Tables

**Table 1:** Detailed information of the four selected sampling sites in Hangzhou.

Sampling Sites	Functional Type	Architecture Type	Vehicle and Personnel Flow	Vegetation Coverage
TJCR	Heavy traffic intersection	High and low office buildings, hotels, and, main traffic road	Approximately 180 vehicles per minute, and 30 persons per minute	Less than 5 percent
ZJGSUJC	Cultural and educational area	Experimental buildings, classrooms, student dormitory, and office buildings around	Few vehicles and approximately 10 persons per minute and the number of about 100 persons per minute off class	About 50 percent
YRBS	Commercial area and business district	Mall and many shopping buildings around	Approximately 60 vehicles per minute and 80 persons per minute	Less than 5 percent
BLQG	Scenic tourist area	No buildings around	Few vehicles and personnel	More than 95 percent

**Table 2:** Types and abundance of airborne bacteria at Family taxonomic levels before and after heat events.

Bacterial Groups	YRBS		BLQG		ZJGSUJC		TJCR	
	-0722 (%)	-0817 (%)	-0722 (%)	-0817 (%)	-0722 (%)	-0817 (%)	-0722 (%)	-0817 (%)
Acetobacteraceae	0.90	0.80	0.62	0.10	0.77	1.79	0.76	1.29
Acidobacteria	0.30	0.42	2.01	0.14	0.50	2.30	0.41	0.65
Aerococcaceae	0.19	0.15	0.08	0.01	0.01	0.10	0.24	0.26
Alcaligenaceae	0.02	0.03	0.81	0.00	0.04	0.01	0.04	0.06
Alcanivoracaceae	0.00	0.00	1.94	0.01	0.00	0.00	0.00	0.00
Alteromonadaceae	0.02	0.03	0.07	0.01	0.00	0.01	0.02	0.05
Bacillaceae	2.86	4.09	1.20	1.10	0.50	6.71	1.38	11.51
Bradyrhizobiaceae	0.25	0.10	1.12	0.05	1.42	0.24	2.32	0.10
Burkholderiaceae	4.95	13.76	3.24	26.79	19.96	8.23	4.11	12.03
Caulobacteraceae	7.37	0.41	3.50	0.06	16.11	0.96	44.69	0.51
Chitinophagaceae	0.34	0.56	1.04	0.07	0.85	1.28	0.50	0.52
Comamonadaceae	0.83	2.87	1.19	3.38	2.88	2.21	1.21	2.40
Corynebacteriaceae	1.24	1.37	0.25	0.12	0.65	0.81	0.51	0.76
Cytophagaceae	1.31	0.41	0.33	0.09	0.55	0.82	0.48	0.36
Deinococcaceae	8.50	4.07	1.22	0.18	2.83	4.19	3.58	7.00
Dermabacteraceae	1.26	0.82	0.20	0.02	0.04	0.24	0.20	0.16
Dietziaceae	0.30	0.20	0.18	0.04	0.03	0.13	0.08	0.34
Enterobacteriaceae	0.12	0.48	0.44	0.08	0.21	0.26	0.13	0.20
Erythrobacteraceae	0.89	0.75	0.17	0.01	0.40	0.50	0.16	0.37
Flavobacteriaceae	0.65	1.11	1.04	0.12	0.40	0.88	0.64	0.48
Geodermatophilaceae	1.18	0.20	0.07	0.00	0.32	0.50	0.16	0.19
Hyphomicrobiaceae	0.39	0.15	0.47	0.02	0.92	0.27	1.96	0.14
Intrasporangiaceae	1.09	0.75	0.19	0.00	0.03	0.27	0.23	0.58
Kineosporiaceae	0.17	0.17	0.09	0.00	0.20	0.75	0.18	0.15
Lactobacillaceae	0.54	0.85	0.15	0.08	0.15	0.38	0.29	0.91
Legionellaceae	0.00	0.03	1.17	0.00	0.01	0.01	0.02	0.02
Methylobacteriaceae	1.92	0.92	0.91	0.87	1.24	1.53	0.84	1.04
Microbacteriaceae	3.17	1.26	1.09	0.15	1.10	2.34	2.19	1.03
Micrococcaceae	9.76	2.79	0.71	0.11	0.29	1.09	0.53	0.99
Moraxellaceae	1.75	3.61	0.84	1.68	2.07	1.57	1.18	2.51
Nocardiaceae	0.10	0.09	1.09	0.02	0.13	0.07	0.21	0.20
Nocardioidaceae	1.36	0.89	1.65	0.13	1.32	2.84	1.97	0.89
Oxalobacteraceae	0.76	0.92	0.48	0.11	0.75	1.04	0.42	1.31
Propionibacteriaceae	1.99	1.25	0.51	0.23	0.24	1.42	0.52	1.15
Pseudomonadaceae	0.77	1.46	2.81	1.13	1.09	1.28	0.49	1.29
Rhodobacteraceae	5.41	2.33	1.03	0.11	1.29	2.68	1.69	1.97
Rhodocyclaceae	5.16	23.40	2.38	58.34	16.49	13.30	3.45	24.62
Rhodospirillaceae	0.32	0.23	4.61	0.02	0.37	0.56	0.27	0.25
Sphingomonadaceae	4.93	1.80	10.90	0.64	6.19	7.53	6.71	3.36
Staphylococcaceae	1.36	2.13	0.28	0.16	0.36	0.91	0.57	0.69
Streptococcaceae	0.47	0.73	0.16	0.03	0.12	0.27	0.14	0.33
Streptomycetaceae	0.77	0.08	0.05	0.02	0.04	0.62	0.24	0.17
Xanthomonadaceae	0.49	0.62	5.43	0.24	4.47	0.48	1.72	0.58
Others	23.85	20.94	42.28	3.53	12.65	26.59	12.55	16.60
